# An optimized framework for quantitative magnetization transfer imaging of the cervical spinal cord in vivo

**DOI:** 10.1002/mrm.26909

**Published:** 2017-09-16

**Authors:** Marco Battiston, Francesco Grussu, Andrada Ianus, Torben Schneider, Ferran Prados, James Fairney, Sebastien Ourselin, Daniel C. Alexander, Mara Cercignani, Claudia A.M. Gandini Wheeler‐Kingshott, Rebecca S. Samson

**Affiliations:** ^1^ Queen Square MS Centre, UCL Institute of Neurology University College London London United Kingdom; ^2^ Centre for Medical Image Computing, Department of Computer Science University College London London United Kingdom; ^3^ Champalimaud Neuroscience Programme, Champalimaud Centre for the Unknown Lisbon Portugal; ^4^ Philips Healthcare Guilford Surrey United Kingdom; ^5^ Translational Imaging Group, Centre for Medical Image Computing, Department of Medical Physics and Biomedical Engineering University College London London United Kingdom; ^6^ UCL Department of Medical Physics and Bioengineering University College London London United Kingdom; ^7^ CISC, Department of Neuroscience Brighton & Sussex Medical School Brighton Sussex United Kingdom; ^8^ Department of Brain and Behavioural Sciences University of Pavia Pavia Italy; ^9^ Brain MRI 3T Mondino Research Center, C. Mondino National Neurological Institute Pavia Italy

**Keywords:** spinal cord, quantitative magnetization transfer, myelin, protocol optimization, reduced field‐of‐view

## Abstract

**Purpose:**

To develop a framework to fully characterize quantitative magnetization transfer indices in the human cervical cord in vivo within a clinically feasible time.

**Methods:**

A dedicated spinal cord imaging protocol for quantitative magnetization transfer was developed using a reduced field‐of‐view approach with echo planar imaging (EPI) readout. Sequence parameters were optimized based in the Cramer‐Rao‐lower bound. Quantitative model parameters (i.e., bound pool fraction, free and bound pool transverse relaxation times [ $T_{2}^{F}$, $T_{2}^{B}$], and forward exchange rate [*k*_FB_]) were estimated implementing a numerical model capable of dealing with the novelties of the sequence adopted. The framework was tested on five healthy subjects.

**Results:**

Cramer‐Rao‐lower bound minimization produces optimal sampling schemes without requiring the establishment of a steady‐state MT effect. The proposed framework allows quantitative voxel‐wise estimation of model parameters at the resolution typically used for spinal cord imaging (i.e. 0.75 × 0.75 × 5 mm^3^), with a protocol duration of ∼35 min. Quantitative magnetization transfer parametric maps agree with literature values. Whole‐cord mean values are: bound pool fraction = 0.11(±0.01), $T_{2}^{F}$ = 46.5(±1.6) ms, $T_{2}^{B}$ = 11.0(±0.2) µs, and *k*_FB_ = 1.95(±0.06) Hz. Protocol optimization has a beneficial effect on reproducibility, especially for $T_{2}^{B}$ and *k*_FB_.

**Conclusion:**

The framework developed enables robust characterization of spinal cord microstructure in vivo using qMT. Magn Reson Med 79:2576–2588, 2018. © 2017 The Authors Magnetic Resonance in Medicine published by Wiley Periodicals, Inc. on behalf of International Society for Magnetic Resonance in Medicine. This is an open access article under the terms of the Creative Commons Attribution License, which permits use, distribution and reproduction in any medium, provided the original work is properly cited.

## INTRODUCTION

Magnetization transfer (MT) refers to the process through which pools of hydrogen nuclei characterized by different molecular environments exchange magnetization. Since its discovery 1, the MT effect has been exploited in MRI as an indirect method for investigating the macromolecular component of biological tissues (e.g., myelin in the central nervous system [CNS]).

Protons attached to macromolecules cannot be probed using conventional MRI because of their ultrashort transverse relaxation time (on the order of microseconds). On the other hand, these protons are sensitive to off‐resonance irradiation because of their broad range of resonance frequencies. Selective saturation of such protons (with off‐resonance pulses) will produce the so‐called MT effect, the transfer of saturation via chemical exchange, and dipole–dipole interactions between the bulk of MR visible free water protons and macromolecular protons, resulting in a signal intensity attenuation in the acquired images.

Typically, the MT effect is measured by the magnetization transfer ratio (MTR), obtained by intensity normalization of an MT‐weighted image with a non‐saturated one 2. Quantitative magnetization transfer (qMT) imaging approaches have been also developed to take into account experimental and biological parameters involved in the MT effect through explicit mathematical modelling 3.

qMT relies on fitting an appropriate model of the acquired signal to a series of MT‐weighted images, to obtain a set of indices related to specific biological features. Various models of the MT‐weighted signal have been proposed over the years 4, 5, 6. While they make use of different approximations to derive analytical expressions and perform differently in relation to noise level and acquisition protocol 7, they can be presented under a unified view by recalling the tissue model they are based on and the spectrum of information they provide.

Most qMT models are based on a two‐pool description of biological tissues consisting of a pool of mobile water protons (i.e., free pool F) and a pool of protons that are bound to macromolecules (i.e., bound pool B). Both pools are characterized by their own relaxation times T_1_ and T_2_ and are thought to exchange magnetization. qMT techniques require the knowledge of the observed longitudinal relaxation time, $T_{1}^{\text{obs}}$, to estimate properties of the two pools. These include each pool's transverse relaxation time ( $T_{2}^{F,B}$), the rate of magnetization exchange from F to B (*k*_FB_), and the relative size of the bound pool or bound pool fraction (BPF). These parameters have proven valuable in assessing myelin integrity in the central nervous system, enabling sensitive examination of macromolecular tissue content without confounds, such as non‐physiological parameters and sequence design specifications, compared to the MTR 8, 9, 10, 11.

The spinal cord is a primary location of demyelination and axonal loss in a variety of diseases, such as multiple sclerosis 12, 13, 14, amyotrophic lateral sclerosis 15, spinal cord injury 16, and neuromyelitis optica 17. Post mortem studies have demonstrated focal and diffuse abnormalities in cord white matter and grey matter in these conditions 12, 14, 18, 19, 20. The development of MRI methods to sensitively look at myelin changes in the spinal cord is therefore an urgent need to provide better explanation of clinical symptoms, to improve the accuracy of current prognosis, and to enable the assessment of emerging neuroprotective or reparative treatments. Hence, qMT methods are of particular interest for spinal cord imaging, although so far the technique has mainly been applied in the brain 21, 22, 23, 24, 25.

The translation to the spinal cord has proven challenging for several reasons: the demands of high‐resolution (to depict spinal cord structure) and, at the same time, adequate signal‐to‐noise ratio (SNR) images to robustly carry out quantitative model fitting result in prohibitive qMT protocol lengths, unfeasible in clinical practice. Furthermore, quantitative MRI of the spinal cord is hindered by high susceptibility to motion artefacts and physiological noise 26, 27.

There are only a few studies that have carried out qMT examinations in the spinal cord in vivo 28, 29, 30, 31, where different solutions (e.g., inversion recovery based qMT or single‐point qMT) have been considered in the attempt to translate qMT methods from the brain to the spinal cord. These approaches are very diverse in nature, rely on several assumptions, or have as yet only been conducted in form of preliminary feasibility studies. As a result, qMT model parameter characterization in the spinal cord is fragmentary, and the agreement between results in literature is only partial.

In this work, we propose a novel framework to foster the implementation of qMT in the spinal cord in vivo, tackling the whole chain, from pulse sequence design to signal modelling and optimization of the sampling scheme, to enable robust assessment of qMT model parameters in acceptable scan times. In particular, an MT‐weighted reduced field of view (rFOV) echo‐planar imaging (EPI) sequence is combined with a dedicated model for unbiased parameter estimation. The sampling scheme is optimized via Cramer‐Rao‐lower‐bounds (CRLBs) minimization, and the reproducibility of qMT metrics is demonstrated in a cohort of healthy volunteers at the cervical level. This framework will easily adapt to other situations where rFOV may be beneficial for assessing indices sensitive to macromolecular components of tissues.

## METHODS

The novel framework, consisting of sequence and signal model developments and protocol optimization, is described below and tested through simulations and in vivo experiments.

### Sequence Design

MT‐weighted images were acquired using an MT‐prepared zonally magnified oblique multi‐slice EPI (ZOOM‐EPI) sequence 32, implemented without using outer volume suppression pulses 33.

**Figure 1:**
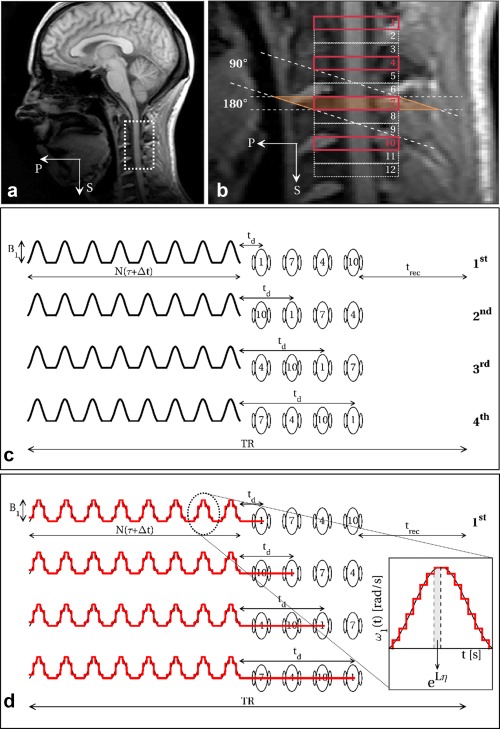
Portion of spinal cord imaged in the sagittal view (**a**), with details of the prescribed slices with ZOOM‐EPI (**b**). Outlined in bold (1, 4, 7, 10) are slices belonging to the same package, that are acquired within the same TR. Slice order within a package is shuffled over different sequence repetitions (**c**), resulting in different delays *t*_d_ between train of pulses and slice excitation. If a number of sequence repetitions that is a multiple of *N*_spp_ (*N*_spp_ = *N*_s_/*N*_p_, *N*_s_ = number of slices, *N*_p_ = number of packages) is prescribed, images can be reconstructed from the average of all slice order configurations, resulting in a homogeneous weighting among different slices. Sequence parameters (*N*, B_1_, τ, Δ*t, t*_d_, and offset frequency Δ) are accounted for in a quantitative setting by an adequate modelling procedure that iteratively solves the two‐pool model Bloch equation (Eq. (2)) through the exponential matrix formalism, using a constant piecewise approximation (discretization step η = 100 µs) for the time‐dependent function ω_1_(t) describing the off‐resonance saturation (**d**).

ZOOM‐EPI 34, 35 allows multi‐slice imaging of small structures using a single‐shot EPI readout. Slices are acquired in an interleaved order, allowing a time interval between contiguous slice acquisition (TR) long enough for longitudinal magnetization to recover following each non‐collinear excitation/refocusing spin‐echo pulse pair. If *N*_s_ is the total number of prescribed slices, this results in *N*_p_ groups (i.e., packages) of *N*_spp_ = *N*_s_/*N*_p_ maximally spaced out slices acquired every TR (Figs. 1a,b).

MT sensitization is achieved via a train of off‐resonance radiofrequency (RF) pulses preceding each package acquisition. In this configuration, *N*_spp_ slices experience the same MT pulse train as they are acquired sequentially following a single train (Fig. 1c). As a consequence, the delay *t*_d_ between the end of the off‐resonance saturation and each slice excitation is dependent on the slice order of the package. To homogenize MT‐weighting across slices, the acquisition is repeated *N*_spp_ times, reshuffling the slice order within each package and averaging the slice signal obtained from each sequence repetition (Fig. 1c). By doing so, slices are reconstructed with homogeneous MT‐weighting and benefit from increased SNR following signal averaging. The same shuffling mechanism is used for the acquisition of the non‐MT‐weighted image used for signal normalization before model fitting, therefore compensating for any potential slice‐dependent off‐resonance effect induced by the excitation/refocusing of neighboring slices. Acquisition parameters are given in the “In Vivo Imaging” section.

### Signal Model

Traditional MT acquisitions in the steady‐state regime would require the use of long trains of MT pulses (>2 s) 3, 4, 5, 6. To exploit the separation of MT preparation from image acquisition for time‐efficient protocols, trains of pulses have to be shortened. As a consequence, a steady‐state MT saturation cannot be established.

The numerical model based on the coupled Bloch equations 36 can be adapted to predict the signal acquired with the sequence described above and estimate fundamental model parameters, accounting for the non‐steady‐state condition.

The model integrates the two‐pool Bloch equations describing the evolution of the three components (x, y, and z) of the magnetization of both pools undergoing exchange and saturation. Given the extremely short $T_{2}^{B}$, transverse components of bound pool magnetization can be discarded. Using the same formalism adopted in 37, two‐pool Bloch equations can be given in the form of homogeneous differential equations, with the following matrix representation:

(1) $$\frac{dM\left(t\right)}{dt}=L\left(t\right)M(t),$$

where **M**(*t*) = ${\left[1/2,M_{x}^{A},M_{y}^{A},M_{z}^{A},M_{z}^{B}\right]}^{T}$, and

(2) $$L\left(t\right)=\left[\begin{array}{ccccc} 0 & 0 & 0 & 0 & 0\\ 0 & \frac{-1}{T_{2}^{F}} & 2\pi \Delta & 0 & 0\\ 0 & -2\pi \Delta & \frac{-1}{T_{2}^{F}} & \omega 1\left(t\right) & 0\\ R_{1}^{F} & 0 & -\omega 1\left(t\right) & -\left(R_{1}^{F}+k_{\text{FB}}\right) & k_{\text{FB}}\frac{1-\text{BPF}}{\text{BPF}}\\ R_{1}^{B}\frac{\text{BPF}}{1-\text{BPF}} & 0 & 0 & k_{\text{FB}} & -\left(R_{1}^{B}+k_{\text{FB}}\frac{1-\text{BPF}}{\text{BPF}}+R_{\text{RF}}^{B}\right) \end{array}\right].$$

Above, Δ is the frequency offset of the MT pulse (in Hz), ω_1_(*t*) = γB_1_(*t*) the time‐dependent amplitude of the MT pulse expressed (in rad s^−1^), characterized by peak amplitude B_1_ (in T) and shape function *s*(*t*) (i.e., B_1_(*t*) = B_1_ *s*(*t*)), $R_{1}^{F}$ and $R_{1}^{B}$ the longitudinal relaxation rates of the two pools, $T_{2}^{F}$ the transverse relaxation time of F, $R_{\text{RF}}^{B}$ the rate of saturation of B (proportional to the super‐Lorentzian absorption line shape 38, dependent on $T_{2}^{B}$), *k*_FB_ the forward exchange rate, and BPF is:

(3) $$BPF=\frac{M_{0}^{B}}{M_{0}^{F}+M_{0}^{B}},$$

where $M_{0}^{F}$ and $M_{0}^{B}$ are the equilibrium magnetizations of the two pools.

The model assumes full relaxation between shots of MT‐prepared ZOOM‐EPI. Within each package, magnetization evolution is predicted by iteratively solving Eq. (2) after replacing the time continuous function ω_1_(t) with an appropriate piecewise approximation, containing the discretized version of the train of MT pulses used (discretization step η = 100 µs) and free precession periods (i.e., when ω_1_(t) = 0) of length *t*_d_ according to the position in the package of the slice currently being acquired, as outlined in Figure 1d.

In addition to the frequency offset Δ, the model explicitly accounts for pulse duration τ, pulse peak amplitude B_1_ (instead of coupling them into the effective flip angle θ), inter‐pulse gap Δ*t*, and number of pulses in the train *N*, which define ω_1_(*t*) over the time period considered in the numerical integration. It also accounts for different delays *t*_d_ resulting from signal averaging while shuffling slices over sequence repetitions.

The model can be fitted to a set of MT‐weighted images to estimate BPF, $T_{2}^{F}$, $T_{2}^{B}$, and *k*_FB_, in combination with a separate measurement of the longitudinal relaxation time $T_{1}^{\text{obs}}$.

### CRLB Optimization

The CRLB theory 39 is applied to derive combinations of sequence parameters **p**_s_ = [B_1_, Δ, τ, Δ*t, N*] that maximize the precision of estimated model parameters **p**_m_ = [BPF, $T_{2}^{F}$, $T_{2}^{B}$, *k*_FB_]. The optimized sampling scheme is defined as the set of combinations of **p**_s_ that minimizes the mean weighted sum of **p**_m_ CRLBs, for a total of *K* measurements and is obtained via minimization of the function:

(4) $$V\left(p_{s,1},\ldots ,p_{s,K},p_{m}\right)=\overset{M}{\underset{i=1}{\sum }}w_{i}\frac{{\left[F^{-1}\right]}_{ii}}{{\left(p_{i}\right)}^{2}}=w_{1}\frac{{\left[F^{-1}\right]}_{11}}{{\left(BPF\right)}^{2}}+w_{2}\frac{{\left[F^{-1}\right]}_{22}}{{\left(T_{2}^{F}\right)}^{2}}+w_{3}\frac{{\left[F^{-1}\right]}_{33}}{{\left(T_{2}^{B}\right)}^{2}}+w_{4}\frac{{\left[F^{-1}\right]}_{44}}{{\left(k_{FB}\right)}^{2},}$$

where [F^−1^]_ii_ represents the *i*‐th diagonal element of the inverse of the Fisher matrix **F**, *p*_i_ is the *i*‐th element of the vector **p**_m_, and *M* the total number of model parameters. The *w*_*i*_ are weights are used to select which model parameter to include in *V*, and therefore assume values *w*_i_ = [0,1].

Knowledge of **p**_m_ is needed in Eq. (4) to solve for optimal **p**_s_. To account for heterogeneity in biological tissue, in practice *V* in Eq. (4) is averaged over *N*_T_ = 6 different plausible tissue configurations **p**_m,n_ (with *N* = 1,…,*N*_T_), taken from previous published works 22, 37, 40, 41.

Optimal sequence parameters are obtained via minimization of the quantity $V\left(p_{s,1},\ldots ,p_{s,K},p_{m,1},\ldots ,p_{m,N_{T}}\right)$, carried out using a self‐organizing migratory algorithm (SOMA) 42, as in Alexander 43.

To reduce the risk of incurring local minima, $T_{2}^{F}$ is excluded from Eq. (4), by setting **w** = [1 0 1 1]. Previous studies have shown that this parameter is characterized by larger variability compared to other qMT parameters 7, 36. However, it does not directly reflect properties of the macromolecular pool and it can be estimated separately with approaches other than qMT, therefore it can be regarded as of minor importance compared to BPF, $T_{2}^{B}$, and *k*_FB_.

Simultaneous optimization of all **p**_s_ could be impaired by the presence of local minima, given the model used (that requires numerical computation). We opted for optimizing only for (Δ, B_1_) pairs, similar to other studies 44, 45, 46. The remaining sequence parameters (τ, Δ*t, N*) are selected with a heuristic approach by comparing a posteriori values of *V* for optimizations at several combinations of (τ, Δ*t, N*). We adopted the following approach: 1 the effect of train length is investigated optimizing for (Δ, B_1_) at different *N* = 10, 20, 30, 40, 50, 60 with fixed τ\Δ*t* = 20 ms\20 ms; and 2 once an optimal train length *N*_opt_ is determined, the effects of τ and Δ*t* are separately tested by running optimization of (Δ, B_*1*_) at different values of τ, with fixed Δ*t* = 20 ms, and vice versa (fix τ = 20 ms), to select τ_opt_ and Δ*t*_opt_. The following values were tested: τ = 10, 15, 20, 30, 40 ms, Δ*t* = 1, 10, 20, 40, 100 ms and 3 final optimization of (Δ, B_*1*_) is carried out with (τ, Δ*t, N*) = (τ_opt_, Δ*t*_opt_, *N*_opt_).

All optimizations were carried out with *K* = 14 sampling points, to produce a protocol consisting of 15 image acquisitions (including one non‐MT‐weighted image), similar to protocols used in the brain. However, the approach can be generalized to a smaller/larger value of *K* to allow for shorter/longer scan times. During optimization, B_1_ was constrained to be below the maximum peak amplitude achievable (13 µT) and simultaneously to avoid SAR deposition above 75% of the maximum allowed value. Frequency offset (Δ) was instead allowed to vary between 1 kHz and 100 kHz. All optimizations were carried out assuming SNR = 25 in the non‐MT‐weighted image, which is plausible for the echo time and resolution used here, given previously reported SNR values with the same readout and instrumentation 47.

To provide a comparison, a non‐optimized protocol, referred to throughout this manuscript as the “uniform protocol,” was also devised. The uniform protocol is designed to resemble standard qMT protocols 21, 40. MT‐weighted data points (a total of *K* as for the optimized protocol) are equally split in two different RF power levels (identified with two distinct B_1_) defined as 80% and 30% of the maximum SAR level allowed in the optimization. At each B_1_ level, Δ are logarithmically spaced between 400 Hz and 20 kHz 21. The same (τ_opt_, Δ*t*_opt_) pair was used for the uniform protocol, whereas to approach the steady‐state condition, which is met in standard qMT experiments, a train of *N* = 50 pulses was chosen, as the maximum length available for the B_1,max_, τ, and Δ*t* selected. Details of the uniform and optimized protocols are given in Table 1.

**Table 1:** MT‐Weighted Sampling Points for the Uniform and Optimal Protocols.

Uniform		Optimal	
Flip Angle (°)	Offset (Hz)	Flip Angle (°)	Offset (Hz)
601	400	378	1018
601	768	383	1031
601	1474	385	1029
601	2828	393	1311
601	5429	426	1706
601	10,420	456	2102
601	20,000	1427	13,710
1100	400	1464	1000
1100	768	1466	3250
1100	1474	1467	3517
1100	2828	1470	3348
1100	5429	1471	3283
1100	10,420	1471	3420
1100	20,000	1471	13,985

MT‐weighted data points are given as effective flip angle and offset frequency pairs. Pulse duration and pulse gap are the same for the two protocols (15 ms/15 ms), whereas pulse train lengths are different (*N* = 50 for the uniform protocol to achieve steady‐state conditions as in previous qMT studies, *N* = 25 for the optimal protocol). The MT pulse shape is sinc‐Gaussian with no lobes.

### Simulations

The efficacy of optimization was tested using Monte Carlo simulations. Synthetic qMT data sets were computed using the optimized and uniform schemes of Table 1. *N*_MC_ = 1000 realizations were generated by adding Rician‐distributed noise at different SNR levels (100, 50, 25, 18, 12).

For each signal realization, one of the *N*_T_ tissue parameter configurations was randomly chosen and perturbed (perturbations were sampled from normal distributions with standard deviation of 0.02, 0.01 ms, 1 µs, and 0.4 Hz for BPF, $T_{2}^{F}$, $T_{2}^{B}$, and *k*_FB_, respectively).

Simulated signals were fitted with the model described in the “signal model” section and percentage errors on model parameters calculated. All model parameters were fitted, and the same $T_{1}^{\text{obs}}$ used for generating the signal was used in the fitting.

Additional simulations were carried out to investigate the effect of errors in pulse amplitude B_1_ and frequency offsets Δ (i.e., B_0_) on parameter estimates for both the optimized and uniform protocols.

### In Vivo Imaging

Five healthy volunteers (3M/2F, 27‐ to 40‐year‐old) were scanned. One volunteer underwent repeated scans (three times) in separate sessions, within 5 days. All volunteers gave informed consent and the study was approved by the local research ethics committee.

Imaging was carried out on a 3T Philips Achieva system (Philips Healthcare, Best, the Netherlands). The full protocol consists of both optimized and uniform qMT acquisitions and an inversion‐recovery (IR) acquisition for $T_{1}^{\text{obs}}$ estimation, shared between qMT protocols.

MT data acquisition was carried out with the MT‐ZOOM‐EPI sequence (see “sequence design”) with: FOV = 48 × 39 mm^2^; in‐plane resolution 0.75 × 0.75 mm^2^; *N*_s_ = 12 axial 5‐mm thick slices centered at the C2/3 disk level; echo time = 28 ms; partial Fourier imaging factor = 0.6. *N*_spp_ = 4 slices were acquired after every off‐resonance pulse train (*t*_d_ = 18, 112, 206, 300 ms) resulting in a TR of 7786 ms and 7037 ms, and a total duration of 23:44 min and 21:27 min for the uniform and optimized protocols, respectively.

T_1_ estimation was carried out using an IR sequence making use of the same ZOOM‐EPI readout (and therefore sharing the same geometry as the MT data), as described in 48. Magnetization recovery was sampled at eight inversion times (TI_min_/Δ*t* = 100 ms/350 ms), same FOV, echo time, and signal averages of the MT‐weighted acquisition, TR = 10550 ms, for a total duration of 15:06 min.

Before fitting, motion within modalities was corrected slice‐wise using FLIRT from FSL 49, and the spinal cord was straightened 50, to co‐register the IR and qMT data sets to each other.

To evaluate protocol optimization in vivo, pooled histograms of model parameters were created for uniform and optimized protocols and inter‐subject CVs calculated. Additionally from the repeated data set, a reproducibility figure for each parameter was calculated voxel wise. The reproducibility index of a model parameter *p*_*i*_, *I*(*p*_*i*_), was defined as 51:

(5) $$I\left(p_{i}\right)=1-\frac{1}{2}(\frac{max\left(p_{i}\right)-min\left(p_{i}\right)}{mean\left(p_{i}\right)}),$$

where *max, min*, and *mean* are evaluated over the three experiment repetitions. *I*(*p*_*i*_) spans from 0 to 1, where 1 indicates ideal reproducibility. Differences between optimized and uniform samplings were explored using a Kolmogorov‐Smirnov (K‐S) test for differences between distributions of *I*(*p*_i_) over the whole cord (significance level *P* < 0.05).

## RESULTS

**Figure 2:**
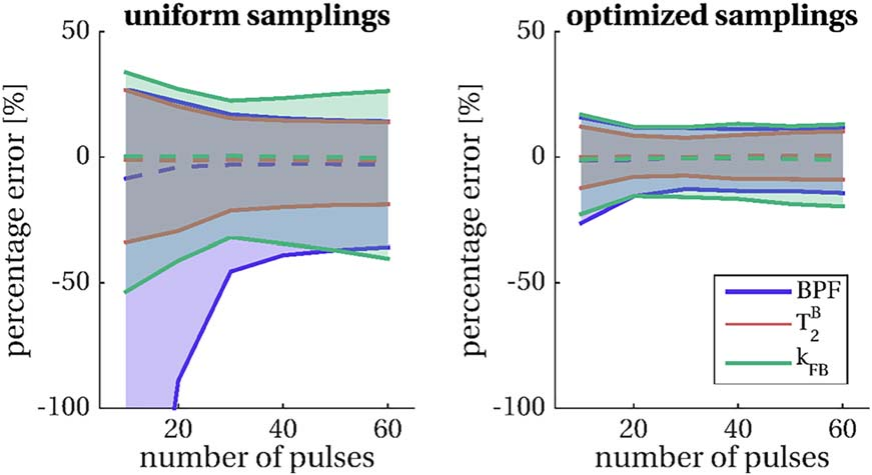
Percentage errors on fitted parameters obtained from Monte Carlo simulations (*N*_MC_ = 1000 repetitions) for uniform sampling (left) and optimal sampling (right) for a varying number of pulses *N* and fixed τ\Δ*t* = 20 ms\20 ms combination. Dashed lines represent the median of error distributions, shaded areas span from the 25^th^ to the 75^th^ percentiles of the distributions. Model parameters considered in the optimization are shown: BPF (blue), $T_{2}^{B}$ (orange) and *k*_FB_ (green). Optimal selection of (Δ, B_1_) pairs reduces parameter errors compared to uniform sampling and greatly mitigates the dependency of the error on the length of the train *N*, allowing the use of shorter, more time‐efficient saturation schemes.

The optimization framework enables the use of non‐steady‐state sequences for accurate fitting of qMT model parameters, as shown in Figure 2. For a given configuration (τ, Δ*t*), errors on fitted parameters can be made almost independent of the length of MT saturation pulse train (Fig. 2b) through adequate selection of sampling points, achieved via CRLB optimization. The example given in Figure 2b shows that a train at *N* = 20 (producing a saturation of 800 ms duration) is comparable in terms of estimation errors to a train at *N* = 60 (of 2400 ms duration). This is in contrast to uniform sampling (Fig. 2a), showing, instead, a strong dependency on *N*. As expected, errors on fitted parameters are reduced in the optimized protocol compared to the uniform protocol.

The length *N* = 25 was identified as the threshold at which parameter errors cease to display dependency on pulse train duration for the given configuration (τ, Δt) and was therefore used as the optimal length *N*_opt_ in the subsequent experiments.

**Figure 3:**
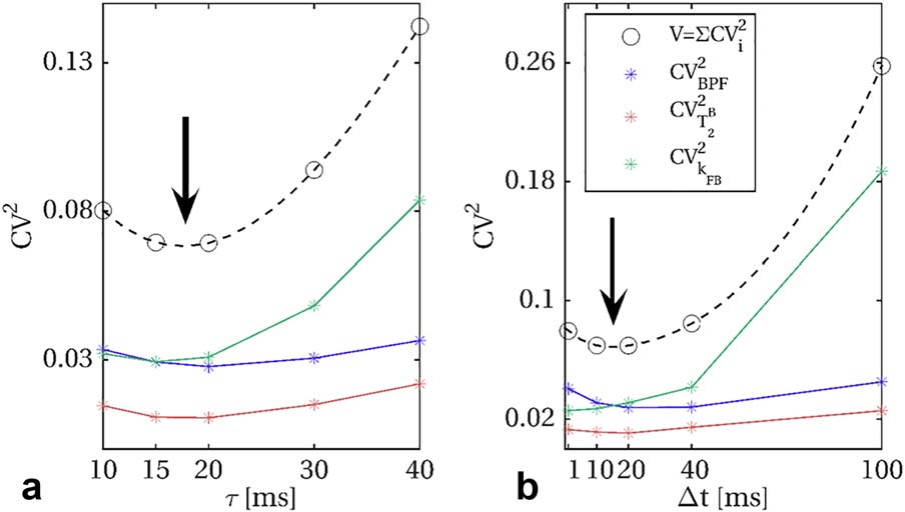
Heuristic search for optimal pulse duration (τ) and pulse gap (Δ*t*), at optimal train length *N*_opt_ = 25. Optimal cost function *V* values for different τ at fixed Δ*t* = 20 ms, and different Δ*t* at fixed τ *=* 20 ms are shown in (**a**) and (**b**), respectively. Spline interpolation between tested configuration is added to the graph (dashed lines), to guide the choice of τ_opt_ and Δ*t*_opt_. The individual contribution of each parameter to the cost function, given by the square of the theoretical CV (obtained from CRLB), is also shown for BPF (blue), $T_{2}^{B}$ (orange), and *k*_FB_ (green). Arrows indicate approximate location of minimal value of *V* as function of the inspected parameters.

Results of the heuristic search for optimal parameters τ_opt_ and Δ*t*_opt_ are shown in Figures 3a,b, respectively. Individual parameter contributions and the total cost function *V* show similar trends in both tests (varying τ at fixed Δ*t*, and varying Δ*t* at fixed τ). Evidence from the combinations tested (τ, Δ*t*) shows that optimal values for both τ and Δ*t* at *N*_opt_ = 25 are between 15 ms and 20 ms. We therefore chose (τ_opt_, Δ*t*_opt_) = (15 ms, 15 ms) as it produces a train of pulses of shorter duration.

Table 1 reports the *K* = 14 optimized pairs (Δ, B_1_) selected by CRLB minimization with τ_opt_, Δ*t*_opt_, *N*_opt_ = 15 ms, 15 ms, 25 together with those defined through uniform sampling with τ_uni_, Δ*t*_uni_, *N*_uni_ = 15 ms, 15 ms, 50. Optimized sampling included points at high B_1_ values, close to the maximum allowed (∼12 µT producing an effective flip angle θ_max_ = 1480 °), and low B_1_ values. Various frequency offsets are selected, between 1 kHz and ∼2 kHz, as well as at higher values (i.e., 13–14 kHz).

**Figure 4:**
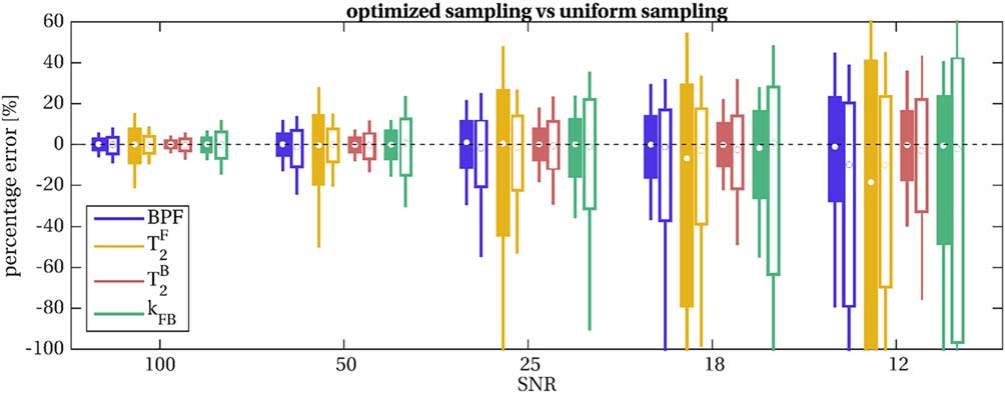
Percentage errors on fitted parameters obtained from Monte Carlo simulations for uniform (unfilled boxplots) and optimal (filled boxplots) protocols at different SNR levels. The optimal protocol produces unbiased and more precise estimates for all the parameters considered: BPF (blue), $T_{2}^{B}$ (orange), and *k*_FB_ (green). Improvements are consistent at every SNR level, including realistic scenarios for spinal cord imaging (SNR < 25). Estimation of $T_{2}^{F}$ is on the other hand more precise for the uniform protocol.

Results from Monte Carlo simulations are shown in Figure 4 for optimized and uniform protocols. CRLB minimization is reflected by a reduction in the variance of parameter errors in simulations, which is consistent at different SNR levels, and becomes more pronounced at lower SNR. Simulations show that improvements are expected for all the model parameters included in the optimization (BPF, $T_{2}^{B}$, and *k*_FB_), with a stronger effect on the exchange rate *k*_FB_. $T_{2}^{F}$ is found more precisely estimated in the uniform protocol than the optimized protocols. However, its inclusion in a further optimization does not improve estimation of the remaining model parameters when compared with the optimized protocol proposed here (Supporting Fig. S4).

Optimized and uniform protocols show a similar dependency on B_1_ errors. On the other hand, the optimized protocol appears more robust to B_0_ errors compared to the uniform one, with distributions of parameters errors within the range (−10%, + 10%) for BPF, $T_{2}^{B}$, and *k*_FB_, for B_0_ variations up to ± 50 Hz (Supporting Fig. S3).

**Figure 5:**
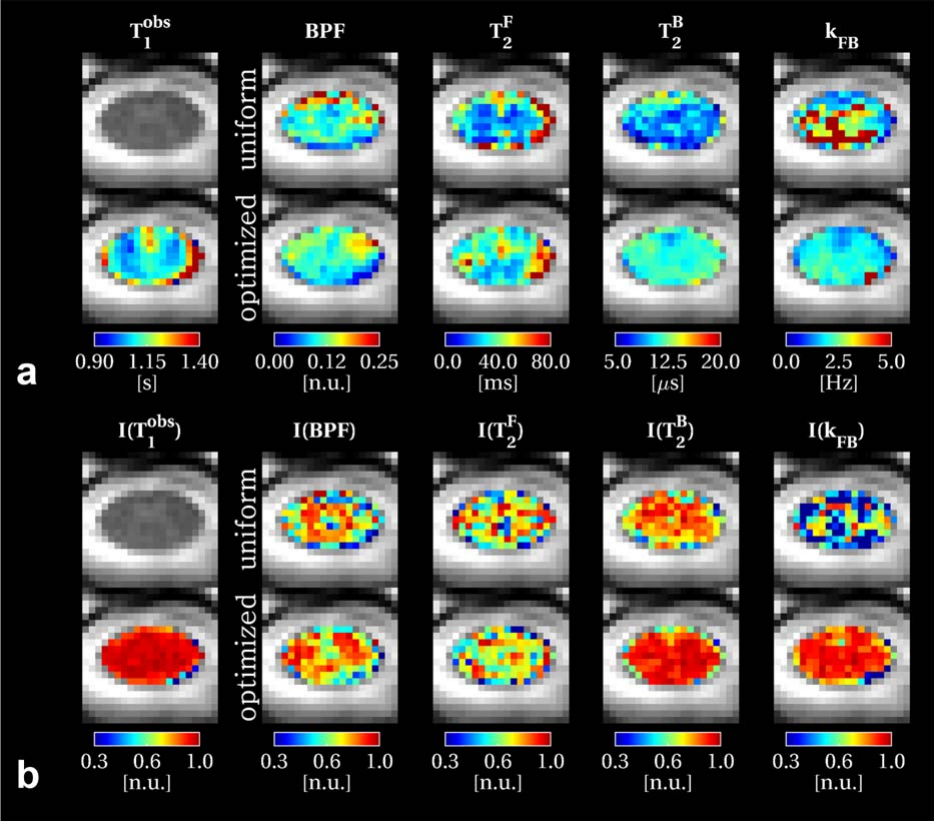
Spinal cord quantitative maps in an example slice. qMT parameter maps are shown in (**a**) both for uniform (top row) and optimized (bottom row) protocols, together with the shared T_1_ maps estimated from the Inversion Recovery protocol. For the same slice, reproducibility indices *I* of model parameters are shown in (**b**). Reproducibility index *I* for a given parameter *p* is calculated from the three repeated acquisition using Eq. (5) and ranges between [0,1] (the higher, the more reproducible the metric is). More examples of qMT parameter maps and reproducibility indices *I* are given in Supporting Figures S5 and S6.

Figure 5a shows parametric maps for both the uniform and optimized protocols for all model parameters from an example slice (more example maps for different subjects are shown in Supporting Fig. S5). Improved spatial homogeneity is visible in *k*_FB_ and $T_{2}^{B}$ maps estimated from the optimized protocol. On the contrary, $T_{2}^{F}$ appears smoother when estimated from uniform sampling. Furthermore, systematic differences can be noticed between uniform and optimized protocol maps. $T_{2}^{B}$ seems to be underestimated in the uniform protocol, confirming the trend seen in simulations shown in Figure 4 at decreasing SNR.

**Figure 6:**
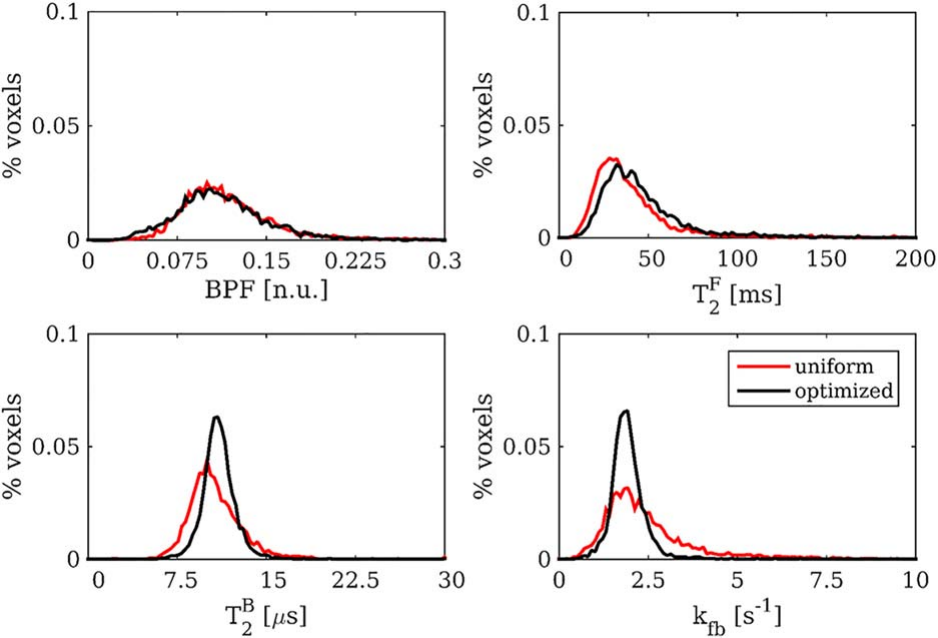
Pooled histograms of model parameters over the cohort of five subjects for uniform (red distributions) and optimal (black distributions). Protocol optimization produces narrower distributions for $T_{2}^{B}$ and *k*_FB_, confirming evidence from the single subject reproducibility study.

Table 2 shows mean and standard deviation for qMT model parameters and $T_{1}^{\text{obs}}$ for each subject, the inter‐subject CV of means, and reproducibility indices calculated voxel wise for the repeated scan over the whole upper cord (levels C1–C4). The effect of the protocol optimization procedure can be straightforwardly appreciated by comparing the standard deviation over the whole cord of parameter estimates, which are substantially reduced for $T_{2}^{B}$ and *k*_FB_ in each subject, as shown by Table 2. Reproducibility indices are shown as parametric maps in Figure 5b, for the same example slice as the model parameter maps in Figure 5a (reproducibility indices over the whole cervical cord are shown in Supporting Fig. S6). The Kolmogorov‐Smirnov test confirmed that $T_{2}^{B}$ and *k*_FB_ were significantly more reproducible for the optimized protocol compared to the uniform protocol (*P* < < 0.05). No difference was detected for BPF reproducibility. $T_{2}^{F}$, although not included in the optimization, showed a statistically significant higher reproducibility (*P* < < 0.05) when using uniform sampling. Figure 6 shows distributions of model parameters for uniform and optimized protocols, pooled among subjects, confirming findings provided by the single‐subject reproducibility test.

**Table 2:** qMT Model Parameters Estimated in the Cohort of Five Subjects for Uniform and Optimized Protocols

Subject	Protocol	Parameters				
		BPF (n.u.)	$T_{2}^{F}$ (ms)	$T_{2}^{B}$ (µs)	*k*_FB_ (s^−1^)	$T_{1}^{\text{obs}}$ (s)
1	*unif*	0.12 (0.04)	38.7 (26.9)	11.5 (3.0)	2.71 (1.54)	1.11 (0.10)
	*opt*	0.12 (0.04)	45.1 (27.0)	11.1 (1.6)	1.88 (0.48)	
	*I*_uniform_	0.74 (0.17)	0.66 (0.23)[Link]	0.83 (0.11)	0.57 (0.27)	0.94 (0.05)
	*I*_optimized_	0.74 (0.16)	0.62 (0.23)	0.87 (0.13)[Link]	0.81 (0.20)[Link]	
2	*unif*	0.11 (0.03)	38.3 (22.0)	10.7 (2.5)	2.41 (1.25)	1.13 (0.12)
	*opt*	0.11 (0.04)	46.7 (21.3)	11.3 (1.9)	1.95 (0.66)	
3	*unif*	0.13 (0.05)	36.7 (21.0)	11.1 (2.5)	2.20 (1.30)	1.15 (0.10)
	*opt*	0.12 (0.05)	44.6 (27.2)	10.6 (1.4)	2.04 (0.75)	
4	*unif*	0.10 (0.03)	46.6 (26.0)	9.9 (2.3)	2.50 (1.17)	1.14 (0.10)
	*opt*	0.10 (0.03)	49.1 (21.9)	11.0 (1.0)	1.90 (0.52)	
5	*unif*	0.12 (0.04)	43.0 (28.8)	10.4 (2.6)	2.56 (1.38)	1.14 (0.16)
	*opt*	0.11 (0.03)	46.9 (25.9)	11.1 (1.7)	1.99 (0.53)	
Mean (SD)	*unif*	0.12 (0.01)	40.7 (3.6)	10.7 (0.6)	2.47 (0.17)	1.13 (0.01)
	*opt*	0.11 (0.01)	46.5 (1.6)	11.0 (0.2)	1.95 (0.06)	
*CV*_intersubj_ (%)	*unif*	7.37	8.93	5.27	6.78	1.03
	*opt*	7.31	3.36	2.14	2.87	

Whole cord mean and standard deviation (in parenthesis) are reported. Means and standard deviations of the reproducibility index, calculated using Eq. (5), are also shown for Subject 1.

^a^Refers to significantly improved reproducibility as measured by the Kolmogorov‐Smirnov test (*P*‐value < 0.05) on distributions of I over the whole cord for either the uniform or optimal protocol when compared to one another. Inter‐subject mean and CV are given at the bottom.

## DISCUSSION

We have developed a framework for qMT experiments in vivo in the cervical spinal cord that minimizes the number of assumptions in the analysis. The major challenges limiting spinal cord qMT applications to date include the need for high‐resolution data to depict spinal cord in detail, the acquisition of enough data points to accurately and reproducibly estimate all the model parameters (BPF, $T_{2}^{F}$, $T_{2}^{B}$, and *k*_FB_) and $T_{1}^{\text{obs}}$, and the need to keep the overall protocol duration within clinically acceptable limits. The framework we propose allows these challenges to be tackled with higher flexibility than solutions that have been investigated so far.

Spinal cord coverage and in‐plane resolution needs are addressed by the use of the ZOOM‐EPI readout, which has previously been successfully applied for spinal cord 34, 52, also in combination with advanced models 47, 53. Time‐efficient generation of MT‐weighting is achieved by adding a train of off‐resonance pulses before the acquisition of a package, exploiting the intrinsic constraints TR > > T_1_ of the ZOOM‐EPI sequence. Such a scheme allows the acquisition of a single MT‐weighted data point in ∼20 s, for the typical cervical cord coverage and resolution used in this study (without signal averaging).

Two main features, specific to this approach, are introduced regarding the MT‐weighting: 1 a time dependency (i.e., the length of the off‐resonance saturation), and 2 a spatial dependency (i.e., the slice position within a package).

With this configuration, steady‐state acquisitions (i.e., with the use of trains of pulses of the order of seconds) would compromise the claimed time efficiency of the sequence. CRLB optimizations, though, clearly demonstrate that even if MT‐weighting depends on pulse train length, the effect the latter has on model parameter estimates is greatly reduced when MT‐weighted sampling points are optimized, resulting in similar performances between trains of different *N*.

In the proposed sequence, MT‐weighting varies among slices within the same package, as these are collected sequentially following the same preparation train (i.e., an increasing effect of T_1_ relaxation is expected to reduce MT‐weighting for slices acquired later on), which will introduce bias in the analysis if not properly addressed. However, the slice order can be shuffled in each sequence repetition to homogenize MT‐weighting across different data points 54, 55. Shuffling can also be carried out within signal averaging repetitions, provided that the number of averages is a multiple of *N*_spp_, resulting in homogenous MT‐weighting across slices for each MT‐weighted data point. We chose this latter solution when designing the qMT protocol for spinal cord imaging.

The additional degrees of freedom in the acquisition (*N* and *t*_d_) are accounted for by implementing an appropriate model, first described by Portnoy and Stanisz 36 and further developed for in vivo qMT in the brain 37. This model was essential to achieve unbiased parameter estimates for images acquired before steady‐state is established (short train of pulses) and during transient evolution of the magnetization (different *t*_d_), as shown in Figure 4 where width of error distributions is minimal at high SNR independently from the type of protocol used.

Furthermore, normalizing the MT‐weighted signal by a reference image obtained with the same slice‐shuffling mechanism provides an inherent correction for the additional MT‐weighting arising from the multi‐slice acquisition module used after the MT preparation, which could be up to 8% of the full signal for the particular sequence used in this study (see Supporting Fig. S1). The difference between model predictions and the simulated signal when accounting for such an effect was always below 0.8% over a wide range of sampling points and tissue parameter configurations (see Supporting Fig. S2).

The framework is integrated with a separate T_1_ measurement obtained from an IR sequence adopting the same ZOOM‐EPI readout used for MT‐weighted acquisition. In such a way, the co‐registration step is greatly improved, given similarities between the two data sets (also in terms of EPI‐like distortions). This is essential to minimize error propagation into qMT parameters caused by potential registration errors with estimated T_1_ maps. Similarly, the choice of ZOOM‐EPI to carry out qMT examination enables images with additional contrast, such as diffusion‐weighted images, to be acquired in the spinal cord in the same fashion for further multi‐parametric analysis. Furthermore, the development of qMT with a rFOV approach could prove beneficial in other challenging imaging environments, such as cardiac, prostate, optic nerve, and musculoskeletal imaging.

The numerical model used here, although introducing a considerable computational burden, gives full control on sequence parameters, which we try to exploit through protocol optimization techniques. qMT protocol optimization has already been investigated in previous studies 44, 45, 46, 56, where sampling schemes were optimized by selecting Δ and θ using standard analytical models. Here, we considered a more general MT model and used CRLB theory to optimize Δ and B_1_, while remaining sequence parameters (*N*, τ_opt_, Δ*t*_opt_) were selected by searching for their best combinations. We cannot disregard the possibility that the heuristic approach followed to determine (τ_opt_, Δ*t*_opt_, *N*_opt_), could lead to suboptimal protocols. Ideally, a simultaneous optimization of all sequence parameters would be preferable, but this would require substantial modifications to the SOMA algorithm to account for the interdependencies between different sequence parameters to be optimized.

An intermediate approach between the heuristic search implemented here and a full optimization of **p**_s_ would be to optimize sampling points split among more configurations of (τ, Δ*t, N*). As shown in Figures 3a,b, expected CVs for individual parameters follow different trends at varying τ and Δ*t*: optimization of BPF tends to favor slightly longer τ and Δ*t*, while *k*_FB_ benefits from shorter pulse repetition time (Δ*t* + τ). Similarly, from Figure 1, BPF errors seem to stabilize at higher *N* compared to *k*_FB_. The single configuration for (τ_opt_, Δt_opt_, *N*_opt_) chosen in this study, based on the trend of the overall cost function value, could have contributed to the lack of clear improvement that we observed on BPF in vivo. Alternatively, protocol optimization could be used to target only a specific parameter 45 by nulling other entries in the weight vector **w**. This could allow the definition of reduced protocols to robustly estimate BPF, while still performing a full qMT model fitting, without introducing any limiting assumptions on other model parameters.

The pattern of optimized sampling points reported in Table 1 shows interesting similarities with previous qMT protocol optimizations using CRLB with analytical models 44, 45. Common features are the presence of repeated points (we counted eight approximately unique points), the sampling at very high Δ (that are likely to produce very little MT saturation), as well as points at the lowest offset allowed (Δ = 1 kHz). The presence of nearly repeated sampling points could be an indicator of the possibility of reducing *K*, and hence the scan time, without sensibly affecting parameter estimates.

The definition of an optimal protocol requires the use of a specific choice of **p**_m_ to compute *V*, suggesting a dependence of the optimal sampling scheme on the set of **p**_m_. We cannot exclude such a dependency in the proposed optimized protocol, however, results from Monte Carlo simulations in Figures 2 and 3 shows that optimization is robust to perturbations on the combinations used in the optimization, as the optimized protocol consistently outperforms the uniform protocol in terms of parameter errors.

Protocol optimization was validated in vivo by computing an index of reproducibility (*I*). This index can be used as a metric to compare optimized and uniform sampling and gain insight into the intrinsic reliability of parameter estimates using the numerical model. The uniform sampling can be taken as an example of a standard qMT protocol, adapted for the sequence developed in this study. Reproducibility indices of qMT model parameters confirm considerations originally shown by Portnoy and Stanisz 36: $T_{2}^{B}$ is the best constrained parameter in the two‐pool model, followed by BPF, $T_{2}^{F}$ and *k*_FB_. Difficulties in estimating the latter two parameters have already been reported 44.

The protocol optimization procedure implemented in this study shows beneficial effects on $T_{2}^{B}$ and *k*_FB_ calculated from in vivo data. Estimation of the latter parameter is particularly improved (*I* increases from 0.57 to 0.81) and its reproducibility is comparable to $T_{2}^{B}$ and higher than BPF. Although the biological meaning of such parameter is not yet fully known, *k*_FB_ has recently received more attention following findings that relate it to inflammation 57 and metabolism 25. Surprisingly, BPF was found to be insensitive to protocol optimization in the in vivo experiment (*I*(BPF) = 0.74 for both uniform and optimized sampling), in contrast to the other model parameters whose reproducibility was significantly affected (*I* is increased for $T_{2}^{B}$ and *k*_FB_ or decreased for $T_{2}^{F}$). As it can be qualitatively appreciated in Figure 5a, and more quantitatively in Figure 6, the optimization procedure also produced systematic differences in parameter estimates, especially in $T_{2}^{B}$ and $T_{2}^{F}$. This has already been observed in a previous study on optimization of qMT parameters 44 and is predicted by simulations reported in Figure 4 that shows an improvement in the accuracy of parameter (included in the optimization) at low SNR. This underlines the importance of implementing protocol optimization techniques when operating at low SNR levels (e.g., for spinal cord imaging).

The reduced reproducibility of $T_{2}^{F}$ in the optimized protocol is a direct consequence of its exclusion from the optimization. However, estimates of BPF, $T_{2}^{B}$ and *k*_FB_ are not affected by a less effective estimation of $T_{2}^{F}$ as shown via simulations in Figure 4 and do not improve when the parameter is included in the protocol optimization (as reported in Supporting Fig. S4). Although estimates of $T_{2}^{F}$ should be considered with caution, especially at low SNR, this approach appears more robust than fixing $T_{2}^{F}$ via constraints, as carried out instead in some previous studies 29, 58.

When compared with previous findings in the spinal cord, summarized in Table 3, qMT parameter estimates lie within the range expected for healthy subjects, with a slightly lower BPF range and slightly higher $T_{2}^{F}$ than previous reported values.

**Table 3:** qMT Parameters Estimates in the Spinal Cord Obtained from the Current Study Using the Optimized Framework and from Previous Studies (Single Values Refer to Whole Cord Instead of WM and GM ROIs)

	BPF (n.u.)		$T_{2}^{F}$ (ms)		$T_{2}^{B}$ (µs)		*k*_FB_ (s^−1^)	
	WM	GM	WM	GM	WM	GM	WM	GM
**1.5T**								
Smith et al. 28	0.12	0.07	NE	NE	9	9	7.84	5.36
**3T**								
Dortch et al. 30	0.18	0.9	24	35.4^a^	11	Fixed	1.71	1.1
Smith et al. 29	0.16	0.14	29.9	32.6^b^	10.8	10.8	1.7	1.46
Smith et al. 29	0.16	0.13	NE	NE	NE	NE	NE	NE
Current study	0.11		46.5		11.0		1.95	
**7T**								
Dortch et al. 31	0.12	0.11	NE	NE	10	Fixed	2.59	1.85

GM, grey matter; NE, not estimated; WM, white matter.

a Estimated from constraint $T_{2}^{F}$ $R_{1}^{F}$ = 0.024, where $R_{1}^{F}$ is fixed to 1 s^−1^ and 0.7 s^−1^ for WM and GM, respectively (from literature).

b Estimated from constraint $T_{2}^{F}$ $R_{1}^{F}$ = 0.0232, where $R_{1}^{F}$ is derived from measured $R_{1}^{\text{obs}}$ equal to 0.806 s^−1^ and 0.752 s^−1^ in WM and GM, respectively.

The spinal cord BPF maps produced here do not provide the typical white matter/grey matter contrast found in the brain (see Supporting Fig. S7). The exacerbated physiological noise characterizing the spinal cord environment, the achievable spatial resolution, which is quite coarse considering the much smaller, detailed anatomy of the spinal cord (with grey matter extending for only a limited number of voxels), as well as potential spatial inaccuracies arising from B_0_ and B_1_ errors surely play a major role in blurring BPF contrast. Aside from technical considerations, assuming that the BPF is mainly associated with myelin, such differences may also be inherently less pronounced compared to the brain, as shown by histological studies 59, 60, where rather uniform intensity maps were observed following staining for myelin.

Through CRLB optimization, we aimed to provide a guide in the definition of sequence parameters for the proposed framework, where additional degrees of freedom in the sampling scheme are available. More work is needed to refine the definition of the acquisition protocol, both to achieve substantial improvement in the estimation of BPF and to reduce the number of the data points *K* without degrading precision of estimates.

Finally, we remark that we did not address in vivo issues related to field inhomogeneities (B_0_ and B_1_). Although these inhomogeneities translate into discrepancies between nominal and actual B_1_ and Δ, and hence inaccuracies in model parameters, especially BPF, $T_{2}^{F}$, and, to a lesser extent, *k*_FB_ (see Supporting Fig. S2), precise characterization of these variations is not straightforward in the spinal cord, and previous studies have reported difficulties in mapping them accurately at the spinal level 61. Additionally, these factors are known to vary smoothly in space and therefore are usually acquired with sequences at coarser resolution (∼3 × 3 mm^2^ in the axial plane) resulting in a limited number of pixels available for their characterization within the cord. These variations are expected to be of a similar size in both optimal and uniform protocols, because both protocols were acquired within the same scanning session. Different sampling patterns can result in different sensitivities of qMT parameters estimates to such errors. The optimized protocol was in fact found to be more robust to errors in Δ than the uniform protocol, most likely caused by the non‐systematic sampling of the offset frequencies. However, improvements in the acquisition strategy to minimize (e.g., via dynamic shimming or slice‐wise shimming) or robustly map these field inhomogeneities are warranted toward an absolute quantification of qMT model parameter in the spinal cord. Similarly, the adaption of the quantitative framework developed here to a cardiac‐gated acquisition should be investigated to minimize artefact from physiological noise that can potentially propagate to parameter estimates.

## CONCLUSIONS

The framework proposed allows robust assessment of qMT model parameters in the cervical spinal cord. The framework includes a dedicated sequence to measure longitudinal relaxation time, is suitable for multi‐modal studies to fully characterize spinal cord microstructure 47, and is applicable to other anatomical environments where rFOV imaging is advantageous. For the first time, parametric maps of qMT model parameters have been shown in vivo in the spinal cord and their reproducibility assessed. Protocol optimization techniques have been used to guide the definition of sampling schemes with the aim of reducing protocol length while improving parameter precisions. Future work will focus on the addition of adequate B_0_ and B_1_ mapping techniques and the possibility to further reduce scan time through more rigorous protocol optimization procedures, as well as combination with further imaging acceleration, such as simultaneous multi‐slice imaging 62.

## Supporting information


**Fig. S1**. Simulations of the effect of off‐resonance saturation caused by a train of on‐resonance spin‐echo in a multi‐slice acquisition, simulated within a package of ZOOM‐EPI. Signal intensity for each slice in the package (numbers 1, 4, 7, 10) is plotted along the rows, whereas each column represents a different sequence repetition, where the slice order is shuffled. The actual slice acquisition order of each repetition is reported at the bottom of each column. Excitation and refocusing pulse shapes, pulse durations, pulse amplitudes and interval between pulses were reproduced in the simulations. The MT effect was simulated using the two‐pool model and results were averaged over 100 combinations of model parameters (randomly sampled from distributions of BPF ∼*N* [0.13%, 0.02%], $T_{2}^{F}$ ∼*N* [46.5 ms, 5 ms], $T_{2}^{B}$ ∼*N* [11 µs, 1 µs], *k*_FB_ ∼*N* [1.95, 0.2], and T_1_ ∼*N* [1.1 s, 0.1 s]). The effect of other slices in the package being off‐resonance during on‐resonance spin‐echo can be visualized for the sequence used in this study. However, given the limited number of slices per package (*N*_spp_ = 4), and the relatively long interval between on‐resonance excitations (Δ*t*_s_ = 91 ms), this additional saturation was found not to exceed 8% of the unsaturated signal.
**Fig. S2**. Simulations of the effect of off‐resonance saturation caused by on‐resonance spin‐echo multi‐slice acquisition on quantitative modelling. MT‐weighting produced by a train of *N* = 25 pulses at five different flip angles (370 ^°^, 650 ^°^, 930 ^°^, 1205 ^°^, 1485 ^°^) for 30 offset frequencies, logarithmically spaced between 500 Hz and 20 kHz, is shown in red. The acquired signal, however, undergoes longitudinal relaxation because of the varying distance between the end of the pulse train and on‐resonance excitation, averaged among different delays *t*_d_ and concomitant off‐resonance saturation because of on‐resonance spin echo (both are dependent on the current slice position within the package). The full MT signal is shown in blue. Before model fitting, MT‐weighted images are normalized to a reference image, M_0_, acquired with the same shuffling strategy. Normalized MT‐weighted signal is shown in black. For quantitative parameters, estimation on resonance‐induced saturation is neglected, and only the effect of averaging between different *t*_d_ is taken in to account. Model predictions are shown by the black dots. It can be appreciated how the normalization with an averaged M_0_ provides a correction for the interslice MT effect (that is inherently present in the normalization term), resulting in only minor discrepancies between the acquired signal and model predictions (average errors over all data points and 100 different tissue configurations is below 0.8%). The normalization corrects for most of the difference between model predictions (no on‐resonance effects) and MT signal (blue line) as shown by the greatly reduced average errors (from ∼4% to ∼ 0.7%). All slices in a ZOOM‐EPI package are shown in different panels.
**Fig. S3**. Effect on qMT model parameters estimates of simulated errors on MT pulse offset frequency (Δ) in (**a**) and MT pulse amplitude (B_1_) in (**b**) for both optimized and uniform protocols (filled and unfilled boxplots, respectively). Errors were introduced by adding a shift in the offset frequency (ΔB_0_ = −200, −100, −50, −20, −10, 10, 20, 50, 100, and 200 Hz) or a scaling factor (ΔB_1_ = 0.8, 0.85, 0.9, 0.95, 1.05, 1.1, 1.15, and 1.2), to the pulse amplitude, respectively, while generating synthetic signals (at SNR = 100). Nominal values for Δ and B_1_ were instead used in the fitting. The optimized protocol appears more robust than the uniform protocol to B_0_ errors, with BPF, $T_{2}^{B}$, and *k*_FB_ error distributions within the −10% to 10% error range for the B_0_ variations expected in the spinal cord (up to 70 Hz). Both protocols appear to be similarly affected by B_1_ errors, with trends replicating previous findings on effect of B_1_ error on qMT model parameters (Boudreau M, Stikov N, and Pike GB. “B_1_‐sensitivity analysis of quantitative magnetization transfer imaging.” Magnetic Resonance in Medicine [2017]; DOI: 10.1002/jmri.25692).
**Fig. S4**. Percentage errors on fitted parameters obtained from Monte Carlo simulations for optimized protocol without including $T_{2}^{F}$ (filled boxplots) and full optimized protocol including $T_{2}^{F}$ (unfilled boxplots) at different SNR levels. The effect of a noisier estimation of $T_{2}^{F}$ does not affect other parameter estimates when sampling schemes are optimized, even at low SNR. Variance of errors on the remaining model parameters is in fact comparable in the two cases, with precision of *k*_FB_ being more effectively improved when optimization does not include $T_{2}^{F}$.
**Fig. S5**. Spinal cord T_1_ (black), BPF (blue box), $T_{2}^{F}$ (yellow box), $T_{2}^{B}$ (orange box), and *k*_FB_ (green box) maps in two example slices from different subjects. For qMT parameters, maps obtained from both uniform and optimal protocol are shown. Greater spatial homogeneity is appreciable in *k*_FB_ maps obtained from the optimal protocol.
**Fig. S6**. Reproducibility index maps for T_1_ (black), BPF (blue box), $T_{2}^{F}$ (yellow box), $T_{2}^{B}$ (red box), and *k*_FB_ (green box) in all the slices acquired (from C1 at the top to C4 at the bottom) for uniform and optimal protocols. Reproducibility index *I* for a given parameter p is calculated from the three repeated acquisition using Eq. (5) and ranges between (0, 1) (the higher, the more reproducible the metric is). Improved reproducibility of parameters with the optimal scheme are found for $T_{2}^{B}$ and *k*_FB_. No differences are detected for BPF, whereas $T_{2}^{F}$ shows higher reproducibility in the uniform protocol. Note also the exquisite reproducibility of the T_1_ estimates obtained with the matched readout inversion recovery sequence used in this study.
**Fig. S7**. Reduced FOV image of the brain displaying WM/GM interfaces, T_1_ maps from inversion recovery, and qMT parameter maps. The identical optimized protocol as that developed for the spinal cord was applied on a localized region of the brain, showing the ability of the framework to differentiate tissue types producing the expected contrast for brain qMT parameters. Specifically, clear contrast in the BPF map between GM and WM can be observed.
