# Supplementary material for: An optimized framework for quantitative magnetization transfer imaging of the cervical spinal cord in vivo
Source: Magn Reson Med. 2017 Sep 16;79(5):2576–88. doi: 10.1002/mrm.26909 (PMC5836910; doi:10.1002/mrm.26909)

**An optimized framework for quantitative Magnetization Transfer imaging of the cervical spinal cord in vivo**

Manuscript # MRM-17-17946

**Supporting information**

Supporting Figure S1

Simulations of the effect of off resonance saturation due to a train of on resonance spin-echo in a multislice acquisition, simulated within a package of ZOOM-EPI. Signal intensity for each slice in the package (numbers 1, 4, 7, 10) is plotted along the rows, whereas each column represents a different sequence repetition, where the slice order is shuffled. The actual slice acquisition order of each repetition is reported at the bottom of each column. Excitation and refocusing pulse shapes, pulse durations, pulse amplitudes and interval between pulses were reproduced in the simulations. The MT effect was simulated using the two-pool model and results were averaged over 100 combinations of model parameters (randomly sampled from distributions of *BPF* ~N(0.13%, 0.02%), *T*_2_^F^~N(46.5ms, 5ms), *T*_2_^B^~N(11µs, 1µs), *k*_FB_~N(1.95, 0.2), and *T*_1_~N(1.1s, 0.1s). The effect of other slices in the package being off-resonance during on-resonance spin-echo can be visualized for the sequence used in this study. However, given the limited number of slices per package (*N*_spp_=4), and the relatively long interval between on resonance excitations (∆*t*_s_=91ms), this additional saturation was found not to exceed 8% of the unsaturated signal.


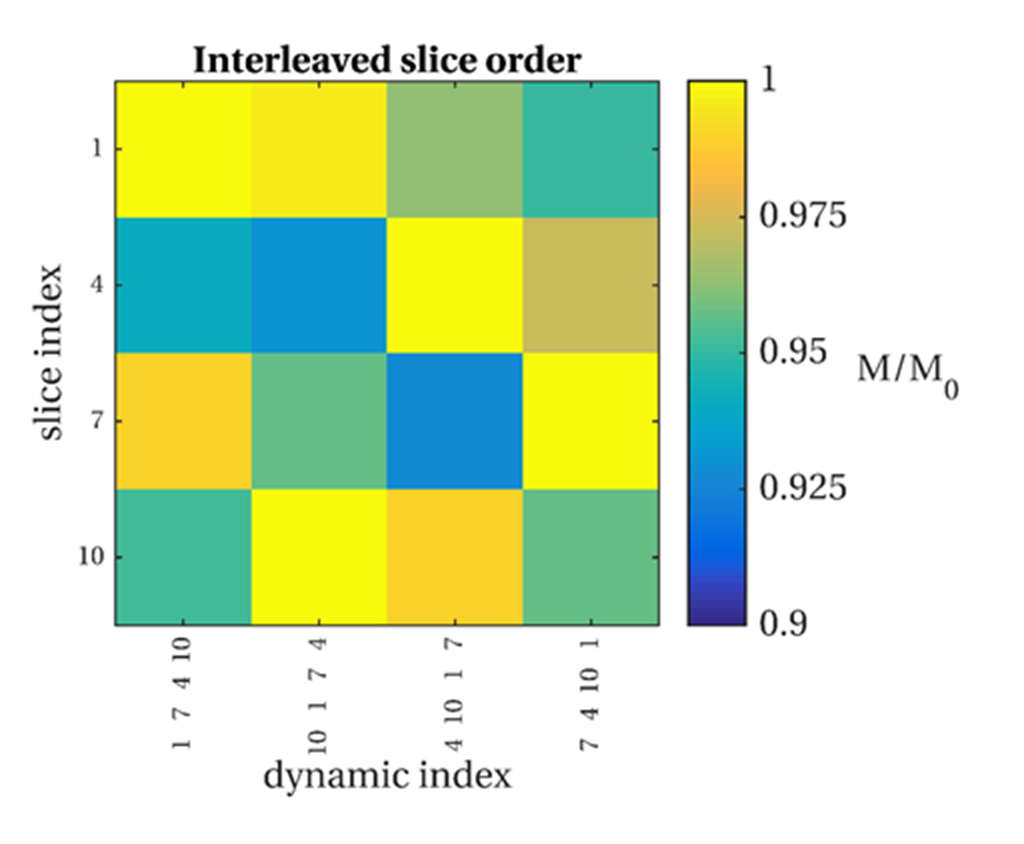


Supporting Figure S2

Simulations of the effect of off resonance saturation due to on resonance spin-echo multi-slice acquisition on quantitative modelling. MT weighting produced by a train of *N*=25 pulses at 5 different flip angles (370°, 650°, 930°, 1205°, 1485°) for 30 offset frequencies, logarithmically spaced between 500Hz and 20kHz, is shown in red. The acquired signal however undergoes longitudinal relaxation due to the varying distance between the end of the pulse train and on-resonance excitation, averaged among different delays *t*_d_ and concomitant off-resonance saturation due to on-resonance spin echo (both are dependent on the current slice position within the package). The full MT signal is shown in blue. Prior to model fitting, MT weighted images are normalized to a reference image, *M*_0_, acquired with the same shuffling strategy. Normalized MT weighted signal is shown in black. For quantitative parameters estimation, on resonance induced saturation is neglected and only the effect of averaging between different *t*_d_ is taken in to account. Model predictions are shown by the black dots. It can be appreciated how the normalization with an averaged *M*_0_ provides a correction for the interslice MT effect (which is inherently present in the normalization term), resulting in only minor discrepancies between the acquired signal and model predictions (average errors over all data points and 100 different tissue configurations is below 0.8%). The normalization corrects for most of the difference between model predictions (no on-resonance effects) and MT signal (blue line) as shown by the greatly reduced average errors (from ~4% to ~ 0.7%). All slices in a ZOOM-EPI package are shown in different panels.


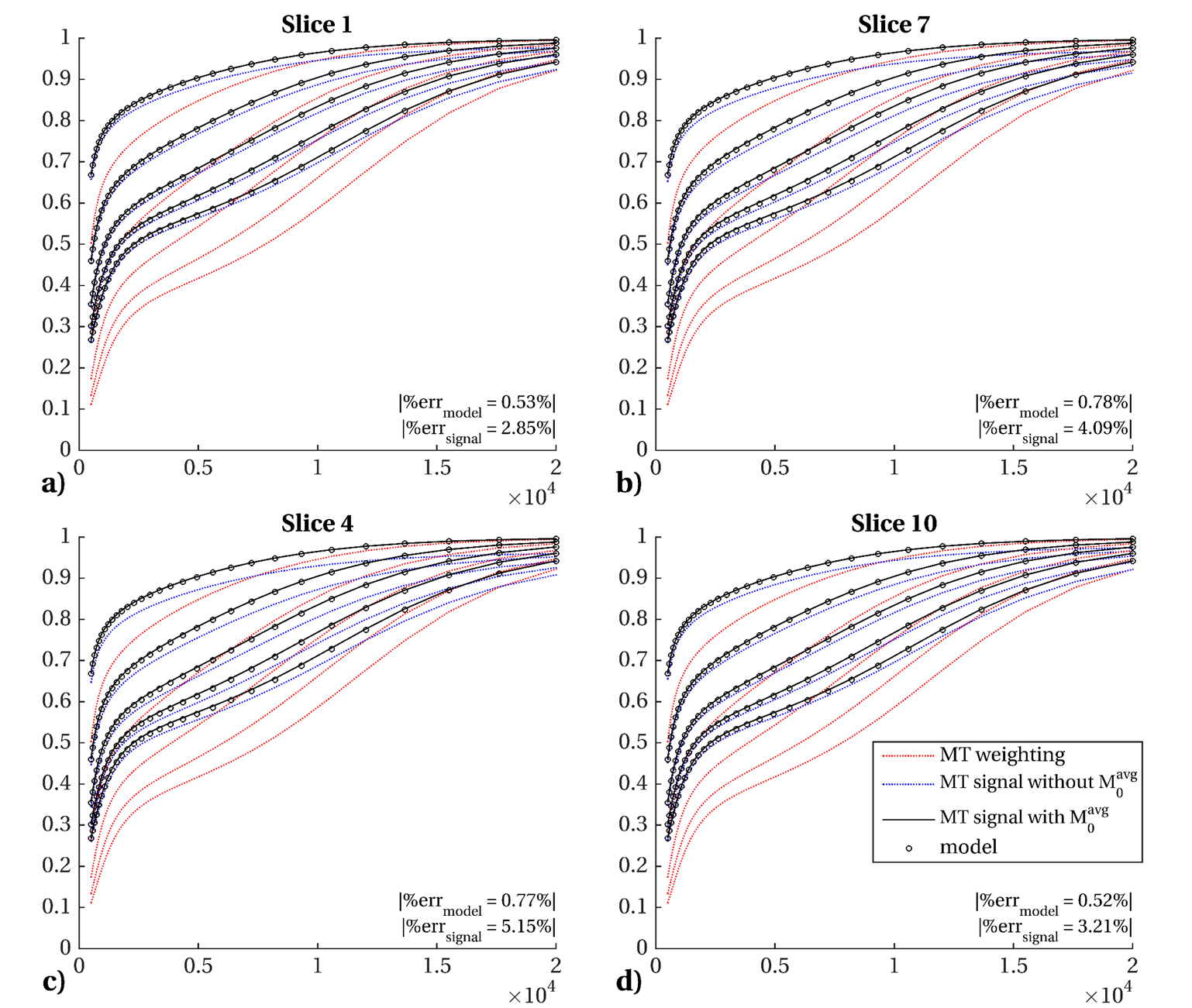


Supporting Figure S3

Effect on qMT model parameters estimates of simulated errors on MT pulse offset frequency (∆) in panel **a**, and MT pulse amplitude (*B*_1_) in panel **b**, for both optimized and uniform protocols (filled and unfilled boxplots, respectively). Errors were introduced by adding a shift in the offset frequency (∆*B*_0_= -200, -100, -50, -20, -10, 10, 20, 50, 100, 200 Hz) or a scaling factor (∆*B*_1_=0.8, 0.85, 0.9, 0.95, 1.05, 1.1, 1.15, 1.2), to the pulse amplitude respectively while generating synthetic signals (at SNR=100). Nominal values for ∆ and *B*_1_ were instead used in the fitting. The optimized protocol appears more robust than the uniform protocol to *B*_0_ errors, with *BPF*, *T*_2_^B^ and *k*_FB_ error distributions within the -10%-10% error range for the *B*_0_ variations expected in the spinal cord (up to 70 Hz). Both protocols appear to be similarly affected by *B*_1_ errors, with trends replicating previous findings on effect of *B*_1_ error on qMT model parameters (Boudreau M, Stikov N, and Pike G B. "B1‐sensitivity
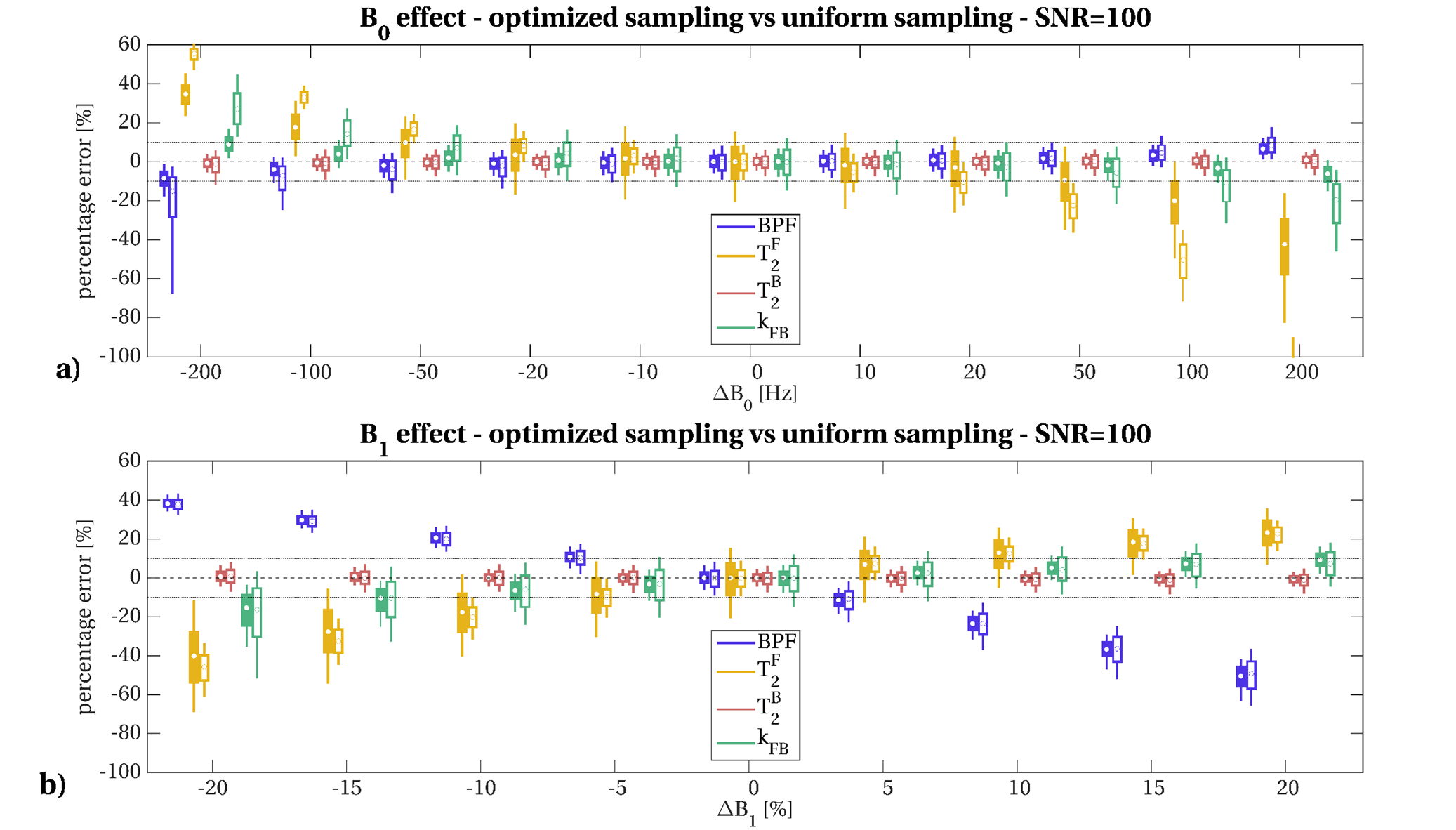
analysis of quantitative magnetization transfer imaging." Magnetic Resonance in Medicine (2017).)

Supporting Figure S4

Percentage errors on fitted parameters obtained from Monte Carlo simulations for optimized protocol without including *T*_2_^F^ (filled boxplots) and full optimized protocol including *T*_2_^F^ (unfilled boxplots) at different SNR levels. The effect of a noisier estimation of *T*_2_^F^ does not affect other parameter estimates when sampling schemes are optimized, even at low SNR. Variance of errors on the remaining model parameters is in fact comparable in the two cases, with precision of *k*_FB_ being more effectively improved when optimization does not include *T*_2_^F^.


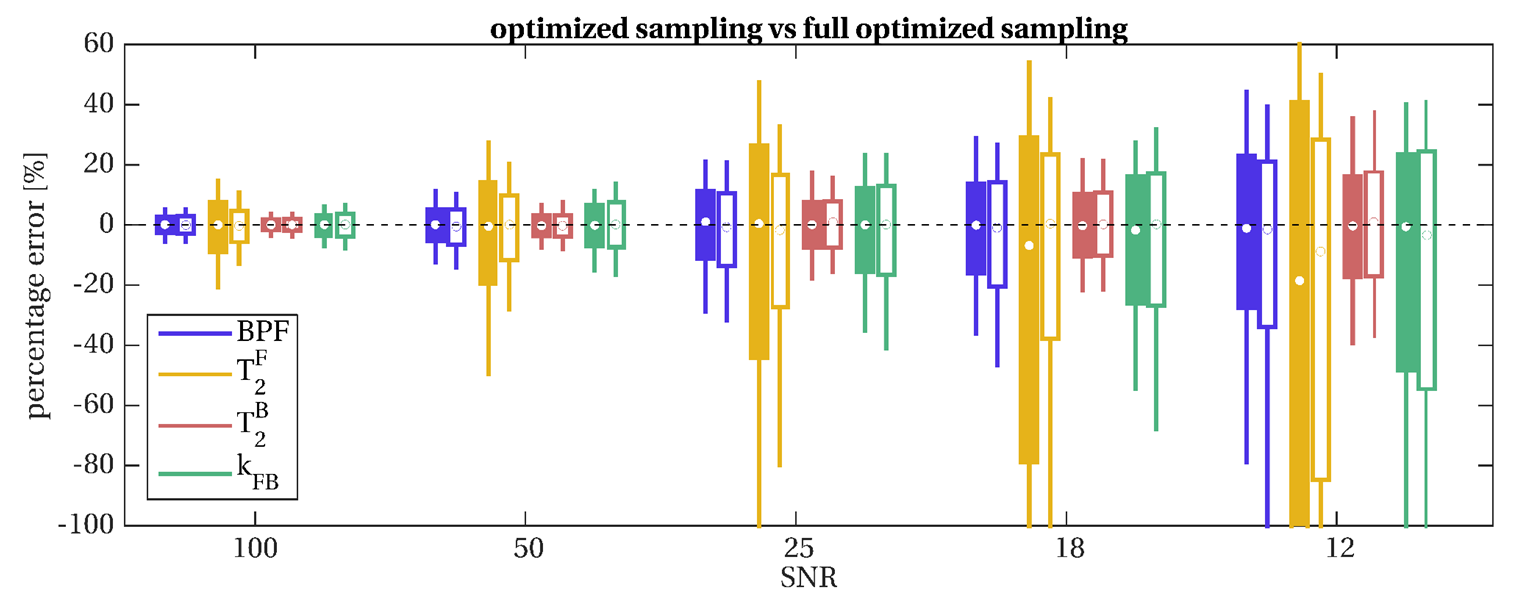


Supporting Figure S5

Spinal cord *T*_1_ (black), *BPF* (blue box), *T*_2_^F^ (yellow box), *T*_2_^B^ (orange box) and *k*_FB_ (green box) maps in 2 example slices from different subjects. For qMT parameters, maps obtained from both uniform and optimal protocol are shown. Greater spatial homogeneity is appreciable in *k*_FB_ maps obtained from the optimal protocol.


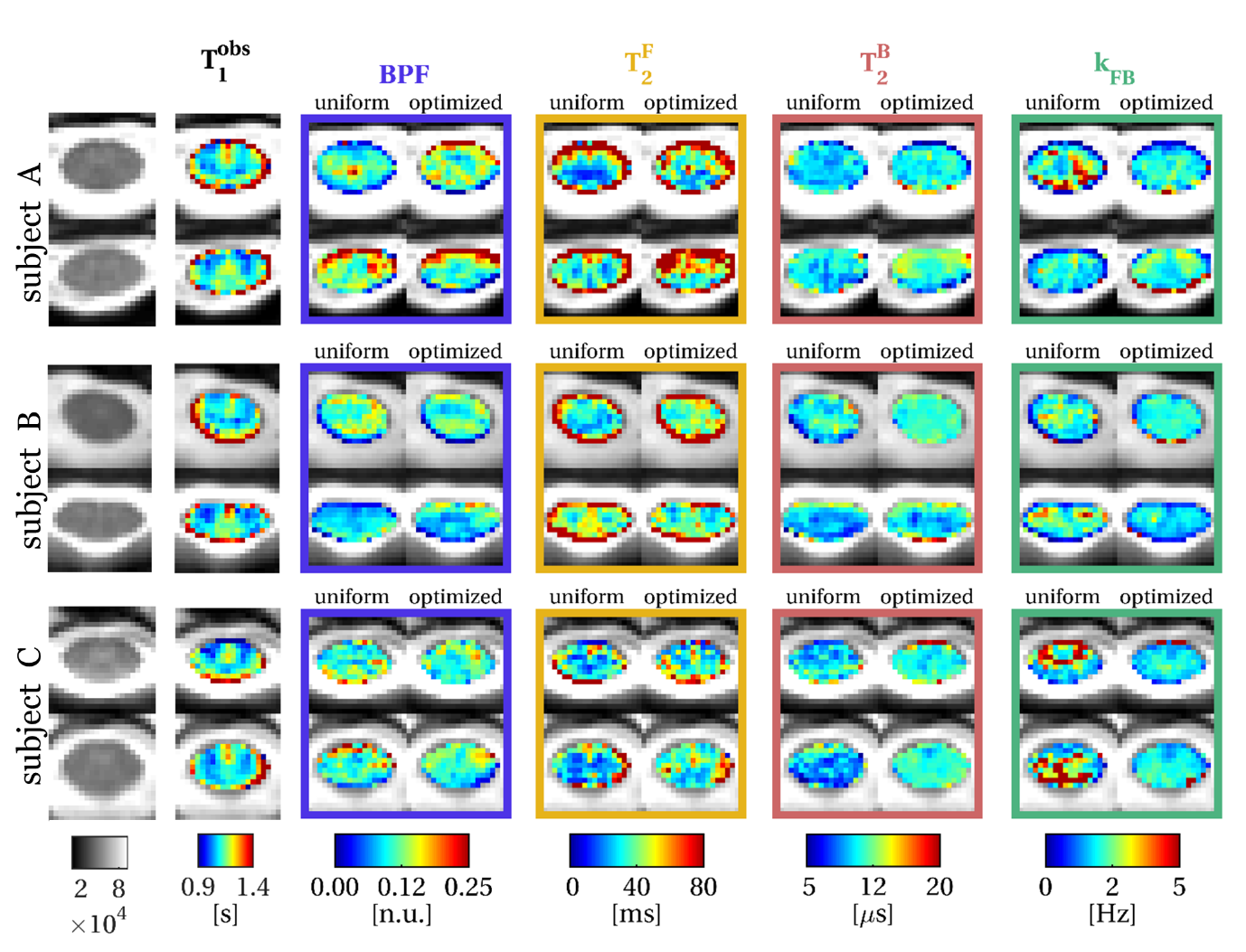


Supporting Figure S6

Reproducibility index maps for *T*_1_ (black), *BPF* (blue box), *T*_2_^F^ (yellow box), *T*_2_^B^ (red box) and *k*_FB_ (green box) in all the slices acquired (from C1 at the top to C4 at the bottom) for uniform and optimal protocols. Reproducibility index *I* for a given parameter p is calculated from the three repeated acquisition using equation 5, and ranges between [0,1] (the higher, the more reproducible the metric is). Improved reproducibility of parameters with the optimal scheme are found for *T*_2_^B^ and *k*_FB_. No differences are detected for *BPF,* while *T*_2_^F^ shows higher reproducibility in the uniform protocol. Note also the exquisite reproducibility of the *T*_1_ estimates obtained with the matched readout Inversion Recovery sequence used in this study.


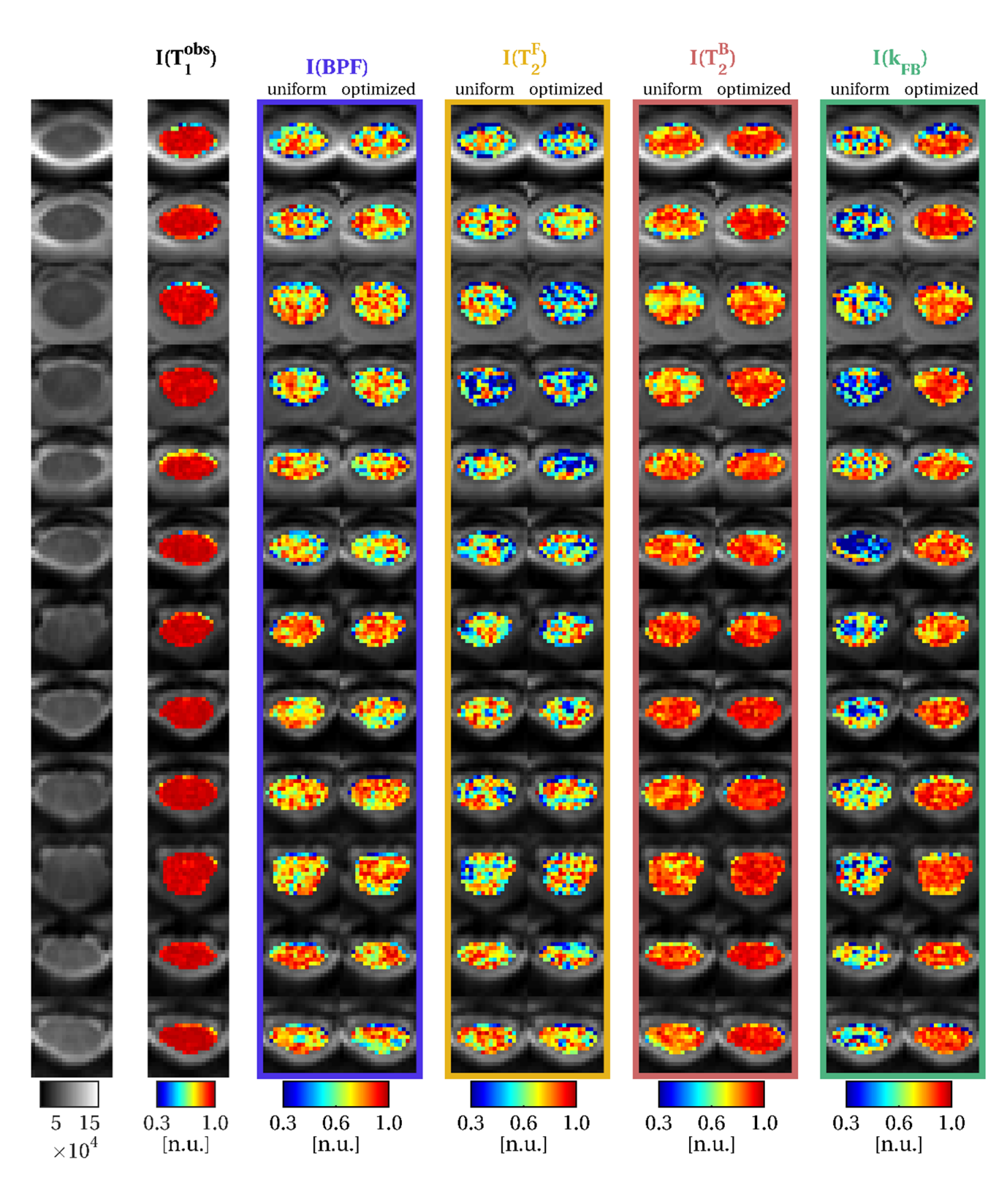


Supporting Figure S7

Reduced FOV image of the brain displaying WM/GM interfaces, T_1_ maps from Inversion Recovery and qMT parameter maps. The identical optimized protocol as that developed for the spinal cord was applied on a localized region of the brain, showing the ability of the framework to differentiate tissue types producing the expected contrast for brain qMT parameters. Specifically, clear contrast in the BPF map between GM and WM can be observed.


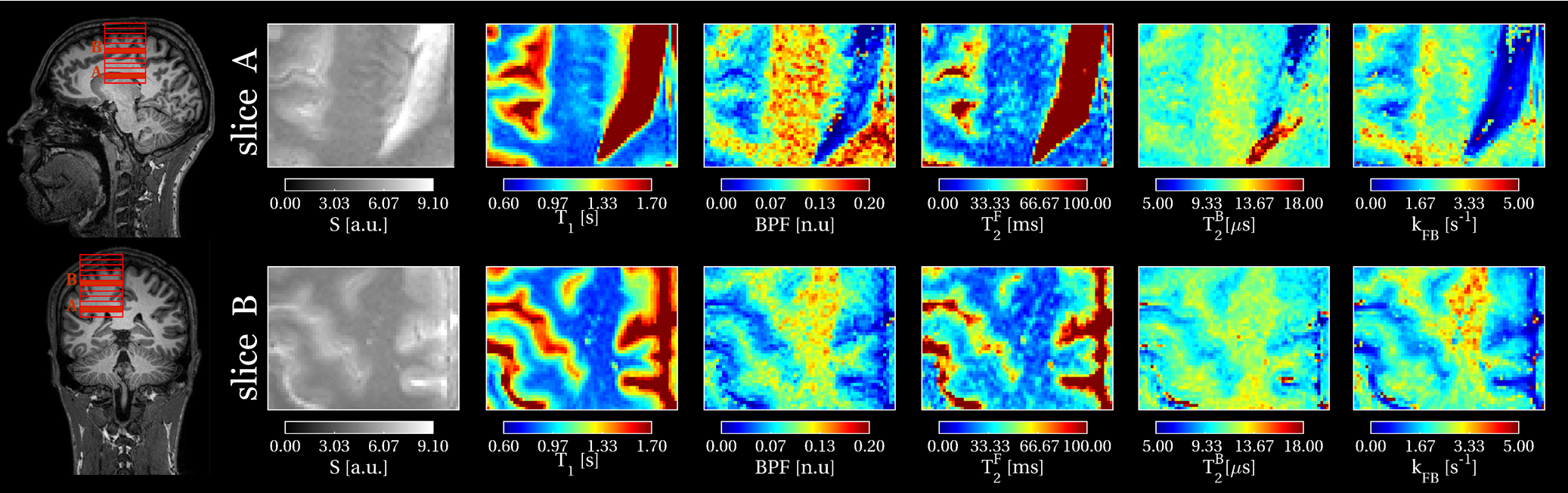

Supplement: Supplementary file 1 — Fig. S1. Simulations of the effect of off‐resonance saturation caused by a train of on‐resonance spin‐echo in a multi‐slice acquisition, simulated within a package of ZOOM‐EPI. Signal intensity for each slice in the package (numbers 1, 4, 7, 10) is plotted along the rows, whereas each column represents a different sequence repetition, where the slice order is shuffled. The actual slice acquisition order of each repetition is reported at the bottom of each column. Excitation and refocusing pulse shapes, pulse durations, pulse amplitudes and interval between pulses were reproduced in the simulations. The MT effect was simulated using the two‐pool model and results were averaged over 100 combinations of model parameters (randomly sampled from distributions of BPF ∼N [0.13%, 0.02%], T2F ∼N [46.5 ms, 5 ms], T2B ∼N [11 µs, 1 µs], k FB ∼N [1.95, 0.2], and T1 ∼N [1.1 s, 0.1 s]). The effect of other slices in the package being off‐resonance during on‐resonance spin‐echo can be visualized for the sequence used in this study. However, given the limited number of slices per package (N spp = 4), and the relatively long interval between on‐resonance excitations (Δt s = 91 ms), this additional saturation was found not to exceed 8% of the unsaturated signal. Fig. S2. Simulations of the effect of off‐resonance saturation caused by on‐resonance spin‐echo multi‐slice acquisition on quantitative modelling. MT‐weighting produced by a train of N = 25 pulses at five different flip angles (370 °, 650 °, 930 °, 1205 °, 1485 °) for 30 offset frequencies, logarithmically spaced between 500 Hz and 20 kHz, is shown in red. The acquired signal, however, undergoes longitudinal relaxation because of the varying distance between the end of the pulse train and on‐resonance excitation, averaged among different delays t d and concomitant off‐resonance saturation because of on‐resonance spin echo (both are dependent on the current slice position within the package). The full MT signal is shown in blue. [file MRM-79-2576-s001.docx]
